# Mad River Dental Society

**Published:** 1873-12

**Authors:** J. A. Stipp, G. W. Keely


### MAD RIVER DENTAL SOCIETY.

The Mad River Valley Dental Society held its regular semi-annual meeting in the parlors of the Phillips House, in Dayton, O., Oct. 7, 1873, commencing at 10 o'clock A. M. President Dr. G. W. Keely in the chair.

Meeting opened with prayer by Dr. A. Berry. Minutes of previous meeting read and approved. Roll called and the following members were present:

Drs. G. W. Keely, S. B. Tizzard, J. A. Stipp, J. Taft, A. Berry, H. A. Smith, R. Corson, D. Gale French, T. K. Brewster, E. F. Sample, R. H. Boal, A. J. Grosvenor, Wm. A. Pease.

The following programme was offered by the Executive Committee:

1.—What obstacles prevent a more rapid development and progress of the dental profession? And how can they be removed?

2.—What are the most urgent demands of the dental profession in its present position?

3.—What means can be employed for the better preservation of the teeth?

4.—What degree of importance attaches to the teeth compared with the other organs of the body?

5.—What assurance is the dentist warranted in giving his patient as to the preservation of his teeth after they are attacked by decay?

6.—What are the modifying circumstances that occasion various results from operations upon the teeth?

7.—How much is the success of filling teeth due to the mere manipulation?

On motion of Dr. T. K. Brewster, the report was received and adopted.

Subjects 1st and 2d were taken up as one, or in connection, and opened by Prof. J. Taft. He remarked that one of the obstacles that prevent a more rapid development and progress in the dental profession, was the want of *discrimination* in the selection of dental students. The qualifications that the student should possess, both natural and acquired, should be examined into. A good English education should be made one of the requirements. And that it was the duty of the dentist to ascertain whether the applicant possessed that; and, if so, have him to resolve attain or, at least, make an effort to acquire the balance. Was of the opinion that in many instances dentists were responsible for so many poor operators.

Dr. A. Berry responded with an amen. Was of the opinion that a dental student was not a very desirable feature in a well-regulated dental office. Yet, if a student was taken, it was the duty of the dentist to examine him early and late in the various branches of professional study, consisting of anatomy, physiology, chemistry and therapeutics.

Dr. G. W. Keely stated that he had taken several students, and in each and every instance had exacted of them close study and diligence. Was proud to say that his success was due to the close application of his students as well as a careful teaching on his part.

On motion, the Society adjourned to meet at 2 o'clock P. M.

AFTERNOON SESSION, 2 O'CLOCK.

Pres. Keely called the Society to order. Discussion of subjects 1st and 2d was resumed.

Dr. H. A. Smith fully concurred with Prof. Taft and others; yet thought that one great obstacle among others was, that dental chemistry was not well understood by dentists at a rge. In regard to dental students believed what had been said to be true. Did not think that the mass of the dental profession was sensible of the present state of affairs, from the fact that they did not mingle with the members in association.

Prof. J. Taft remarked that the majority of students who entered the profession do so with a view of making money; and in many instances were encouraged in that by dentists themselves. The good of humanity should be at heart—make that a consideration, and one of the impediments, at least, would be removed. Another obstacle was the want of appreciation of the means and agencies offered to the profession. Rejecting instruments and appliances essential in making a good operation; clinging to "things" belonging really to ancient times.

Dr. H. A. Smith thought money a good thing to have around. That the dentist should be reasonably compensated for his services. Many times when we have done our duty honestly and conscientiously, and when the bill is presented, a feeling of ungratefulness is manifested.

Dr. T. K. Brewster was of the opinion that the community at large should be educated up to a point of appreciation of a good operator. That they should be made to understand the difference between a *good dentist* and a *quack*.

Prof. J. Taft remarked, in regard to the demands of the profession, that it was the duty of dental societies and dentists generally to make an effort to raise the standing of all to a higher and more honorable position. The community demanded it. The profession, in a measure, were responsible for the men now in the profession.

Dr. H. A. Smith expressed a hope that legislation would do a great deal more for the profession, and that those who would wish to come into it in the future would have to comply with the requirements or stay out of it.

Dr. T. K. Brewster inquired if the dental colleges were not disposed to graduate persons for money, thereby throwing upon the profession men not worthy of a title, and dragging the profession down with them.

Prof. Taft replied that colleges, or at least some of them *were not influenced by money*. That dentists were responsible, by permitting students to attend such institutions when there were colleges in the land that were deserving, and would send out men prepared to practice in the profession.

Miscellaneous :

On motion, a committee was appointed to revise the roll.

Adjourned to meet at 7 1-2 o'clock, P. M.

EVENING SESSION, 8 O'CLOCK, P. M.

Society called to order by Pres. Keely.

The committee appointed to revise the roll submitted their report.

On motion of Prof. Taft, the report was received and adopted.

The Rec. Sec. was instructed to notify members in arrears, and, unless they responded by payment of dues, their names would be stricken from the roll.

Subject 3d was announced.

Prof. J. Taft stated that the object of introducing the subject in this form was to elicit something more than that already known. That it was the duty of the dentist to instruct his patients, under all circumstances, to cleanse the mouth and teeth, and to *keep* them thoroughly cleansed. By so doing, it would assist them in maintaining their general health. In fact, it was the duty of the dentist to impress upon every patient the importance of saving the natural teeth. It should be presented to them in its various phases as an essential to health, beauty and comfort. Pecuniary circumstances should not indicate when and where to instruct them ; experience had taught him in many instances that the poor servant-girl had the highest appreciation of her teeth, and of the labor performed upon them.

Dr. H. A. Smith reported a case in which a lady had suffered severely during gestation. The demands made upon her system had been of such a character as to cause considerable uneasiness in and about the teeth. Explained to her the cause, and prescribed syrup of lacto-phosphate of lime. Would report progress in the future.

Dr. Pease thought that a crowded condition of the teeth would cause trouble during gestation. Used the burr or separating file to obtain space. Thought that Dr. Arthur's method of treatment should be adopted.

Dr. J. A. Stipp asked for information. Could not understand how it was that we recommended food containing an abundance of the phosphates, to assist nature in the *more perfect* development of natural teeth, and then use the burr or separating file to cut away enamel, exposing the dentine, subjecting it to the action of the fluids of the mouth. To him, it looked as though we were tearing down that which we were trying to build up.

Dr. D. G. French fully concurred with Dr. Stipp in regard to Dr Arthur's treatment. Thought if teeth were *well* filled, and proper care taken of them, by frequent brushing, much more would be accomplished. Objected seriously to the burr and file.

Dr. R. H. Boal experiences the same difficulty in the teeth of mothers during gestation. Found the teeth of the child in after years to occasion trouble. Thought that Dr. Arthur's method of treatment would produce evil results.

Prof. J. Taft thought that Dr. Arthur had made quite a mistake, in not making some discrimination. That it had a tendency to lead many into error. As a rule it was not advisable to pursue that course of treatment.

Dr. G. W. Keely spoke of the importance of saving the natural teeth. Urged the frequent use of a good brush, and exercising care in keeping the teeth clean. Does not think Dr. Arthur intended that we should cut away as heroically as suggested in his work. Cuts away only when it seems to be for the best.

Dr. A. Berry thought it to be a retrograde movement, to go back to the old way of cutting and filing away tooth substance. Better try and keep it, as decay takes it away fast enough.

Miscellaneous :

Pres. Keely appointed the following Executive Committee for the ensuing year : Drs. H. A. Smith, S. B. Tizzard and R. H. Boal.

Essayists : Drs. R. H. Boal, S. B. Tizzard and A. J. Grosvenor.

The hour of 12 o'clock having arrived, the Society adjourned to meet in Dayton, O., on the first Tuesday in April, 1874.

J. A. Stipp, *Rec. Sec.*,

G. W. Keely, *President.*

